# Discriminative Learning of Receptive Fields from Responses to Non-Gaussian Stimulus Ensembles

**DOI:** 10.1371/journal.pone.0093062

**Published:** 2014-04-03

**Authors:** Arne F. Meyer, Jan-Philipp Diepenbrock, Max F. K. Happel, Frank W. Ohl, Jörn Anemüller

**Affiliations:** 1 Department of Medical Physics and Acoustics and Cluster of Excellence ''Hearing4all'', University of Oldenburg, Oldenburg, Germany; 2 Department of Systems Physiology of Learning, Leibniz Institute for Neurobiology, Magdeburg, Germany; 3 Department of Neuroprosthetics, Institute of Biology, Otto-von-Guericke University, Magdeburg, Germany; International School for Advanced Studies, Italy

## Abstract

Analysis of sensory neurons' processing characteristics requires simultaneous measurement of presented stimuli and concurrent spike responses. The functional transformation from high-dimensional stimulus space to the binary space of spike and non-spike responses is commonly described with linear-nonlinear models, whose linear filter component describes the neuron's receptive field. From a machine learning perspective, this corresponds to the binary classification problem of discriminating spike-eliciting from non-spike-eliciting stimulus examples. The classification-based receptive field (CbRF) estimation method proposed here adapts a linear large-margin classifier to optimally predict experimental stimulus-response data and subsequently interprets learned classifier weights as the neuron's receptive field filter. Computational learning theory provides a theoretical framework for learning from data and guarantees optimality in the sense that the risk of erroneously assigning a spike-eliciting stimulus example to the non-spike class (and vice versa) is minimized. Efficacy of the CbRF method is validated with simulations and for auditory spectro-temporal receptive field (STRF) estimation from experimental recordings in the auditory midbrain of Mongolian gerbils. Acoustic stimulation is performed with frequency-modulated tone complexes that mimic properties of natural stimuli, specifically non-Gaussian amplitude distribution and higher-order correlations. Results demonstrate that the proposed approach successfully identifies correct underlying STRFs, even in cases where second-order methods based on the spike-triggered average (STA) do not. Applied to small data samples, the method is shown to converge on smaller amounts of experimental recordings and with lower estimation variance than the generalized linear model and recent information theoretic methods. Thus, CbRF estimation may prove useful for investigation of neuronal processes in response to natural stimuli and in settings where rapid adaptation is induced by experimental design.

## Introduction

**Figure 1:**
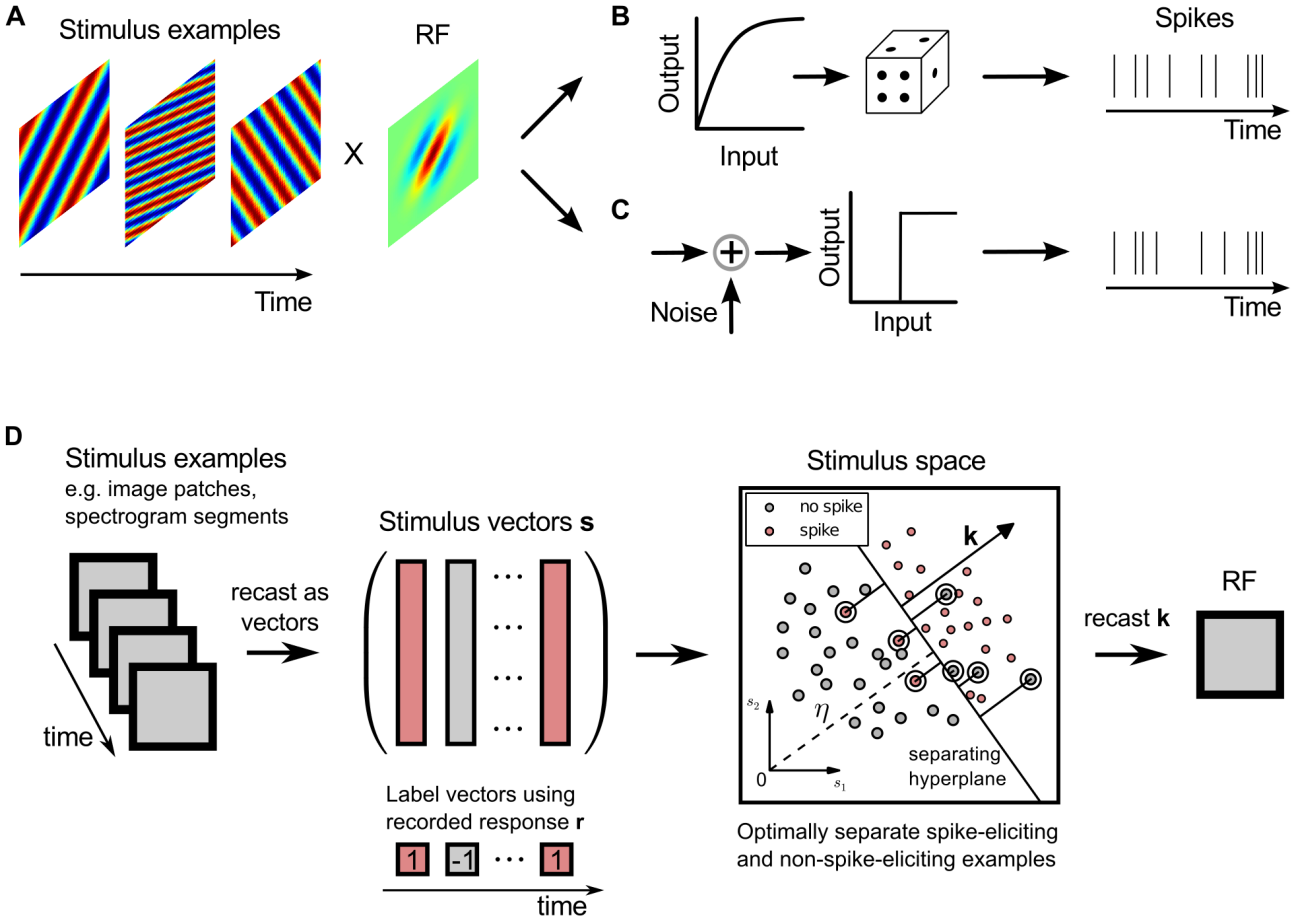
Classification-based receptive field estimation. (**A**) Linear models of neural response generation perform integration of stimulus features using a linear filter corresponding to the neuron's receptive field (RF). (**B**) Standard linear-nonlinear Poisson (LNP) model: a static memoryless nonlinearity is applied to the linear stimulus projection with subsequent Poisson spike generation. (**C**) The binary model assumed here generates spikes from the (noisy) linear projection through an explicit threshold operation. (**D**) Stimulus examples, e.g., image or spectrogram patches, are recast as vectors in order to estimate the linear filter in the binary model. The binary responses recorded in experiments are used to label the resulting vectors as spike-eliciting (red) or non-spike-eliciting (gray). The proposed classification-based receptive field (CbRF) estimation method aims to identify the hyperplane that optimally separates the spike and non-spike classes in a high-dimensional space, whose dimensionality corresponds to the stimulus vectors' dimensionality. The box illustrates the principle in two dimensions: a large-margin classifier adjusts the separating hyperplane with normal vector k such that the risk of misclassifying stimulus examples is minimized. Misclassified stimulus examples are marked by white circles and their distance to the hyperplane by black lines. The estimate of the linear RF filter corresponds to the recast normal vector of the hyperplane.

Characterizing responses to sensory stimuli is fundamental for understanding how biological systems encode information about the outer world into a robust internal representation. At the level of single neurons, information is encoded in a sequence of spike and non-spike events [1], [2]. The way stimuli are encoded in this binary sequence is commonly analyzed using the receptive field (RF), a functional model relating sensory stimulus and evoked response (for a review see [3], [4]). As illustrated in Figure 1 **A** processing in the RF model is performed by a linear projection of stimuli through the neuron's linear filter, and a subsequent nonlinear operation that governs the neuron's spike response (Figure 1 **B**). Such a cascade is also known as linear-nonlinear Poisson (LNP, [5]) model. The linear filter corresponds to the RF of a neuron and describes how that neuron integrates stimulus features. Neural coding in terms of the RF has been applied to different sensory modalities, e.g., in the visual system [6]–[9] and in the auditory system [10]–[16].

However, even in the seemingly simple RF case, estimation is non-trivial since estimation algorithms are not only influenced by the true underlying system parameters, but also by the statistics of the stimulus ensemble [17], [18]. When the stimulus ensemble is composed of stimuli with non-Gaussian distribution or higher-order correlations across stimulus components, linear RF estimation methods like the spike-triggered average (STA, [19]) and derived variants, e.g., [7], [11], [20], [21], may not correctly identify the underlying linear RF parameters [8], [22]. Recently developed information-based estimators allow RF estimation under more general conditions at the expense of optimization procedures that may lead to suboptimal RF estimates, particularly for small sample sizes [8], [18], [23].

The generalized linear model (GLM) framework [24] provides a flexible approach to linear-nonlinear model parameter estimation. A GLM utilizes a linear predictor and an invertible link function to infer the system response's expectation value and probability density. Spike interactions may be incorporated in terms of a post-spike history filter [9], [25]–[27]. For arbitrary stimulus ensembles, the GLM is proven to provide an unbiased estimator of the response if the chosen inverse link function corresponds to the neuronal processing nonlinearity. Thus, a mismatch between hypothesized and actual nonlinearity may lead to biased estimates [25]. Iterative fitting of the linear filter and the nonlinear link function may reduce the bias and provides a numerical approximation to maximization of mutual information between stimulus and response in case the number of spikes is small [23].

Here, a classification-based method is proposed that reliably estimates a neuron's RF when the stimuli possess characteristics akin to those of natural stimuli, involving non-Gaussian statistics and higher-order correlations within the stimulus ensemble. The rationale for the approach is based on the classic notion of the McCulloch-Pitts model [28] in which neurons are regarded as binary decision units that linearly sum inputs and respond with the presence or absence of a spike depending on whether a (possibly noisy) threshold is exceeded or not. Figure 1 **C** illustrates the corresponding generative model in which spikes are generated from projections of stimulus examples onto the linear filter, followed by a noisy threshold operation. The spike threshold, as a fundamental part of the neuron's response, is explicitly accounted for in the model, and the stochasticity in the neuron's response is incorporated through the additive noise term.

To learn the parameters of the model we have to find the classifier parameters such that the probability of falsely detecting spike or non-spike examples is minimized. The principle is illustrated in Figure 1 **D**. A stimulus 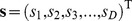, such as the spectro-temporal density of an acoustic stimulus preceding the response, is represented by a vector in a 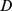-dimensional space. Based on the observed response 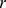, where 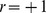 and 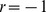 indicate the presence or absence of a spike, respectively, the stimulus is assigned to either the spike or non-spike class. The goal is to find the linear filter 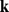 such that spike and non-spike stimulus examples are maximally separated in the 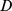-dimensional space. Maximum separation of spike-conditional stimulus examples is directly related to the concept of empirical minimization of the misclassification error [29]. Thus, the optimal 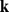 minimizes the risk of falsely predicting a spike or no spike on the data and represents an estimate of the neuron's linear filter.

The underlying optimization corresponds to a classification task and we will refer to this approach as classification-based RF (CbRF) estimation. To find the parameters of the model we propose an algorithm based on a large-margin classifier (see Materials and Methods). We demonstrate that incorporation of spike and non-spike probabilities is required to obtain robust parameter estimates for non-Gaussian stimulus ensembles. The resulting estimator is robust to asymmetric stimulus distributions and second- and even higher-order correlations in the stimulus ensemble. It bears resemblance to maximization of mutual information between stimulus and response in case the fraction of stimuli that evoke a spike is small.

These findings are validated using simulations and recordings from the inferior colliculus of Mongolian gerbils for responses to highly non-Gaussian stimuli. We find that the classification-based method is less sensitive to the detailed form of the nonlinearity than the GLM when probed with natural stimuli. In the large-data regime the proposed approach performs equally to information-theoretic estimators with the benefit of much better convergence properties. Thus, the CbRF method allows robust response characterization, even in situations in which common estimators may not provide reliable estimates of the RF parameters.

## Materials and Methods

### Classification-Based Receptive Field Estimation

#### Binary model of neural coding

In experiments, we present 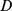-dimensional sensory stimulus examples 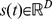 from an ensemble of stimuli while recording the one-dimensional response 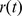 to these examples from a specific neuron. We assume that the response is already discretized and assumes binary values, with 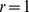 denoting that a spike has been elicited and 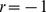 indicating the absence of a spike. In the auditory system, 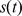 usually contains the spectro-temporal density preceding the response in a specific time window. In the visual system, 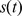 may represent a sequence of image patches.

When a spike is observed at time 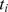 it is assumed that there is some pattern in the stimulus example 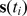 that elicited the spike and is characteristic for that neuron. Intuitively, observing that specific pattern should increase the probability of detecting a spike. In a simplified model, this can be quantified by the projection 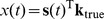 of the stimulus onto the linear filter 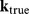 that characterizes feature sensitivity of the neuron. To obtain a binary response a threshold operation is applied to produce a spike if the stimulus example contains a pattern similar to 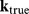 and 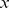 assumes high values. Furthermore, neural responses are not deterministic and we have to account for neural noise.

The time-dependent (binary) response 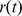 of the system is given by.
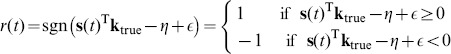
(1)with a noise term 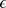 centered around the spiking threshold 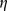 and signum function 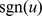, which is 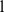 for 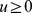, and 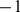 for 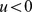. The shape of the corresponding static nonlinearity in the LNP model is determined by the cumulative density function of the neural noise.

#### Estimation of model parameters

Numerical solutions for direct estimation of 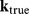 and 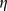 in the binary model (Eq. (1)) from data lead to a non-convex optimization problem and may result in suboptimal estimates. Instead, a convex upper bound to the binary loss is obtained by minimization of the objective function.
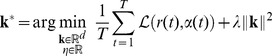
(2)


with loss function 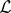, 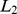-norm regularization term 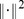, and 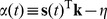. The loss function is a nonlinear function of how distant misclassified examples are from the separating hyperplane in stimulus space (cf. Figure 1 **D**) and thus determines the degree to which "small'' and "large'' errors, respectively, are penalized. The choice of 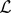 is crucial to find optimal solutions [30], [31]. We use the squared hinge loss.
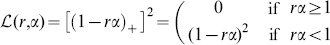
(3)


and 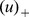 is a function such that 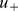 is 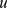, if 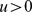, and zero, otherwise. The regularization term effectively maximizes the geometric margin between spike and non-spike class and is controlled by the regularization parameter 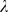, which is found using cross-validation.

The optimization in Eq. (2) corresponds to the general form of a large-margin classifier and does not involve explicit estimation of class probabilities. However, the employed loss function directly aims at the optimal decision rule that minimizes the risk of misclassifying stimulus examples assuming knowledge of the true conditional probabilities 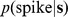 of a spike being generated given stimulus 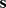 [32]. Thus, if responses were generated according to the noisy threshold model illustrated in Figure 1 **C**, the proposed approach is guaranteed to find the optimal parameters. This is also true for other loss functions; prominent examples are exponential loss, logistic loss, and hinge loss [30], [31], [33]. The latter corresponds to the square root of the squared hinge loss and is closely related to the support vector machine (SVM) methodology, which is motivated by maximizing the geometric margin between classes [34], [35].

The above definition of the problem assumes that spike and non-spike examples occur with equal probability, i.e. 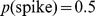 and 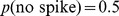. However, spikes are sparse, particularly in cortical areas, making it necessary to extend Eq. (2) to account for highly unbalanced spike and non-spike classes. Prior information may be introduced into Eq. (2) by replacing the loss function 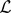 with the weighted loss 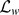,
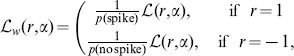
(4)which weights errors of spike and non-spike examples by the corresponding inverse probabilities [33], [36].

**Figure 2:**
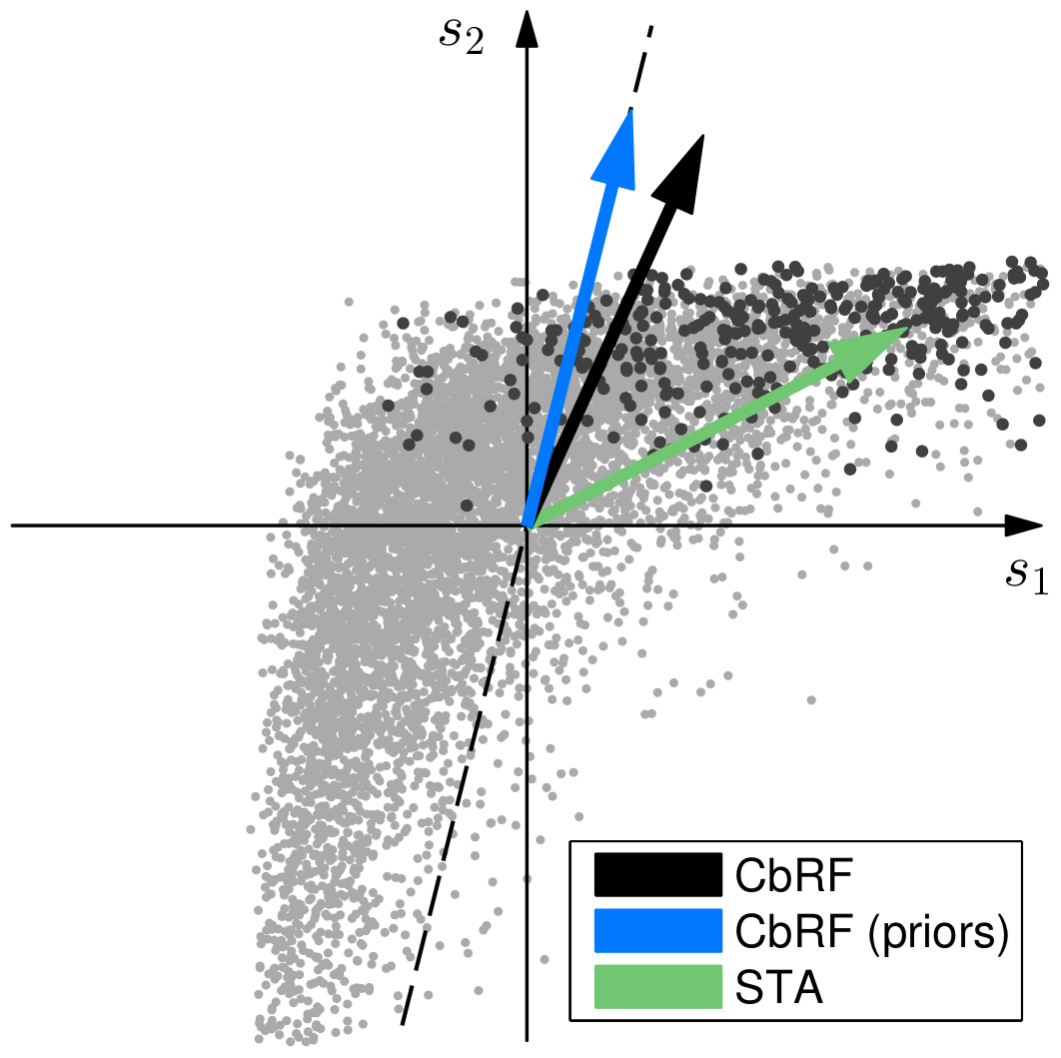
Effect of incorporating class prior probabilities into the objective function. LNP simulation with a 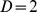-dimensional uncorrelated stimulus, asymmetric stimulus distribution and sigmoid-shaped nonlinearity. The dashed line indicates direction of the ground truth linear filter. Light and dark gray dots represent presence or absence of spikes, respectively, with 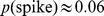. Filter estimates represented by the normal vectors of the decision hyperplanes have been estimated using the proposed classification-based method with true class prior weighting (blue arrow) and uniform weighting (black arrow) of misclassification errors. The green arrow illustrates the spike-triggered average (STA) solution. The filter direction estimate obtained with uniform weighting systematically deviates from the true direction. For visualization purposes, normal vectors of decision hyperplanes have been rescaled equal length.

Figure 2 shows the difference between solutions with and without weighting of misclassification errors for a two-dimensional example with 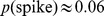. Without weighting, the solution systematically deviates from the true solution, whereas the weighted solution recovers the ground truth RF. For comparison we also tested the linear spike-triggered average (STA) estimator (see Methods S1). The STA solution is highly biased due to violation of the symmetry assumption.

#### Relation between class decisions and conditional distributions

Here, we will show that prior-based weighting of misclassification errors (cf. Eq. (4)) provides a link between spike-conditional projections onto the linear filter and spike-conditional distributions. This interpretation allows to relate the CbRF method to probabilistic approaches, e.g., information-theoretic estimators (see below).

Assume we have estimated linear filter 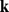 and spiking threshold 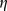 for a given stimulus–response set. After projection onto 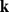, the joint distribution of projected stimuli 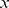 and spike labels 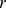 is given by 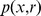. The probability mass in the two slices of this distribution, 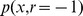 and 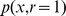, is distributed very unevenly. However, the class-specific weighting proportional to the inverse prior probabilities implies sampling from the (normalized) conditional distributions 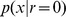 and 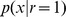 due to the equality.
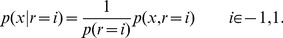
(5)


In consequence, weighted errors obtained in the limit of high and low threshold values, respectively, are equal, reflecting the symmetric influence of the two classes on the binary error function.
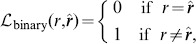
(6)where 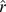 is the response predicted by Eq. (1). This is also true for the CbRF method, which optimizes a convex upper bound to the binary misclassification error.

#### Optimal spike decisions minimize threshold noise

**Figure 3:**
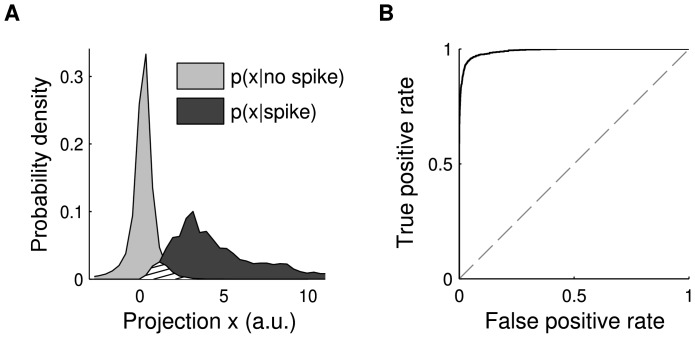
Misclassification risk and threshold noise. (**A**) Distributions of spike-eliciting (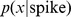; dark gray) and non-spike-eliciting (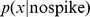; light gray) stimulus examples after projection onto CbRF method-derived linear filter; abscissa in arbitrary model units (a.u.). The hatched area indicates the overlap of the distributions where the model produces non-deterministic classification responses. (**B**) Receiver operating characteristic (ROC) analysis of classification performance for one estimated RF filter, obtained by varying the decision threshold applied to projection 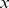 and subsequent plotting of resulting true positive and false positive classification rates, i.e. threshold varies along the curve. The (0, 1) point indicates optimal performance and corresponds to vanishing noise around the threshold. The area under the ROC curve constitutes a measure of the misclassification risk specific to the underlying RF filter and decreases with increasing noise levels. Regularization parameter 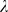 of the CbRF model (Eq. 2) is obtained by maximization of area under the ROC curve during cross-validation on the training data. Results shown based on data from LNP model simulations with natural image stimuli.

Let 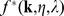 denote a decision rule for which we have estimated linear filter 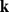 and spiking threshold 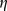 for a particular choice of the regularization parameter 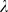. According to Eq. (1), decisions are made by applying a threshold operation to the projections 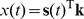 of the spike-eliciting and non-spike-eliciting stimulus examples onto the estimated linear filter. As a result of prior-based class weighting, the distributions associated with the conditional projections, namely 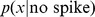 and 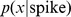, determine the expected misclassification risk. An example for spike-conditional and non-spike-conditional distributions is shown in Figure 3 **A**. In the region where the density of one of the distributions is close to zero the response can be considered to be essentially deterministic. In the transition region, the separability highly depends on the overlap, which is determined by the noise level around the threshold. Therefore, the optimal filter estimate is given by the model that results in the smallest overlap between the distributions, corresponding to the lowest achievable noise level.

A similar approach has previously been used in physiological studies to quantify the discrimination sensitivity of neurons between two possible decisions, e.g., [37], [38]. It is based on the receiver operating characteristics (ROC) curve, which is generated by plotting the fraction of correctly detected spike examples ("true positive rate'') versus the fraction of falsely detected non-spike examples ("false positive rate'') for different spiking thresholds. In the linear threshold model, this is equivalent to "shifting'' the threshold along the axis of stimulus projections and estimating the rates from the distributions. The ROC curve for the example is shown in Figure 3 **B**. The overlap between the distributions can be quantified by integrating over all thresholds yielding the area under the ROC curve (AUC). A value close to 1 corresponds to a small overlap, whereas a value close to 0.5 indicates highly overlapping distributions manifesting in a random response.

A similar scenario occurs in information-theoretic RF estimation that seeks to maximize mutual information (MI) between stimulus and response in a linear-nonlinear model [8], [18], [23]. MI is given by the Kullback–Leibler divergence (cf. Eq. (1) in Methods S1) between prior and spike-triggered stimulus distributions 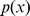 and 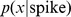, respectively [39]. If the probability of the occurrence of a spike is small, i.e. 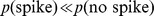, the distribution of non-spike-conditional projections, 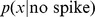, is effectively equivalent to 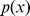, as is usually the case for sensory neurons. Hence, both unconstrained maximization of MI and constrained minimization of the relative misclassification risk aim for models that minimize threshold noise. RF estimation based on MI maximization is known as "maximum informative dimensions'' (MID, see Methods S1 for details) [18]. For comparison, we also applied to MID method to the data.

#### Numerical Optimization

The optimization problem in Eq. (2) is convex and permits efficient solution using standard gradient descent methods. Here we used a Newton conjugate gradient trust-region algorithm for unconstrained minimization [40]. The regularization parameter 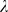 is chosen using five-fold cross-validation on the training set. The value of 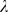 that results in the highest cross-validated AUC is used to estimate the final RF parameters. On a full IC data set determination of the best regularization parameter took less than 10 minutes on a current multi-processor computer.

### Physiology Experiment

#### Ethics statement

All experiments were conducted in accordance with the international National Institutes of Health Guidelines for Animals in Research and with ethical standards for the care and use of animals in research defined by the German Law for the protection of experimental animals. Experiments were approved by an ethics committee of the state Saxony–Anhalt, Germany.

#### Electrophysiology

Recordings in the inferior colliculus (IC) were made in 31 ketamine/xylazine anesthetized adult male Mongolian gerbils (Meriones unguiculatus; age, 3–16 months; body weight, 80–120 g). For detailed description of the surgical procedure and electrophysiological recordings please see [41]. Briefly, single-unit recordings in IC were made with tungsten electrodes (3–4 M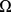) via dorsoventral insertion using a Plexon Multichannel Acquisition Processor (Plexon Inc). Recording within IC was ensured by stereotactic coordinates and tracking down the electrode until short-latent (approx. 6–10 ms spike latency) responses to brief tone-pips were found [42], [43]. Single-unit data was verified off-line using the software Offline Sorter (Plexon Inc). Only units that produced at least 100 spikes per trial have been considered for the analysis.

#### Stimulus generation

We used two stimulus ensembles. The first ensemble was composed of consecutive blocks of frequency-modulated tones. A block with randomly drawn starting and ending frequencies between 0.5 kHz and 16 kHz is generated according to.
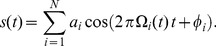
(7)


We set 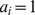 and 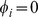, 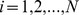, for all 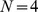 sweeps in a block. The block length is 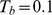 s and 5 ms half-cosine ramps are used at the beginning and at the end of each sweep. The length of the stimulus sequence is 100 s and the whole sequence has been repeated five times. In the the second stimulus ensemble the same FM sweeps started continuously in time under the constraint that the average sweep density is between 3 and 4 sweeps.

In this study, we used linear sweeps, 
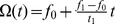
, with starting and ending frequencies 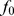 and 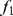, respectively, and 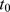 and 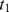 are the corresponding time instants. Sounds were delivered by an amplifier to a calibrated Canton Plus XS.2 speaker and presented free field in a double-walled sound booth. For analysis, the stimuli were transformed into their time-frequency representation by filtering the sound pressure waveform using a gammatone filterbank into octave-like frequency bands (approx. 2 filters per octave). Compression resulting from the cochlea has been simulated by applying log-compression to the envelope of the filter outputs.

## Results

### Receptive Field Estimation from Simulated Responses

#### Robustness to asymmetric stimulus distributions

**Figure 4:**
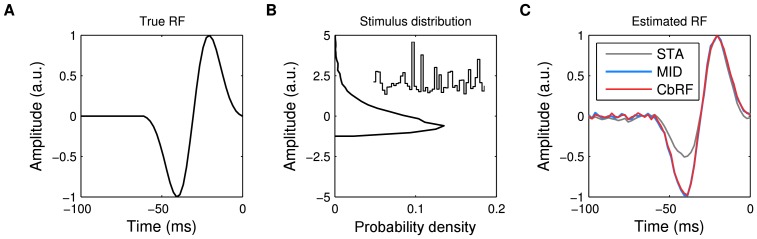
Robustness of RF filter estimation to stimulus distribution asymmetries, obtained with LNP model simulations and asymmetric white noise stimuli. (**A**) Ground truth linear filter underlying the simulations. (**B**) Stimulus amplitude distribution with long tail towards positive values, created by drawing 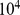 samples from a Gaussian white noise distribution and subsequent expansion (compression) of positive (negative) amplitudes, respectively. The stimulus auto-covariance matrix remains diagonal, simplifying the linear estimator to STA without covariance correction. 50 samples of the temporal stimulus sequence shown in the inlet. (**C**) Estimates of the linear filter obtained using STA, MID and CbRF approach. While the latter two methods reconstruct the true linear filter faithfully, the STA-based estimate shows a non-symmetric scaling of the positive and negative deflection. Linear filter amplitudes rescaled to arbitrary units (a.u.) in the interval 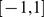 for visualization.

We demonstrate the robustness of the proposed method by considering a model neuron whose RF can be described by a temporal filter. As indicated in Figure 4 **A** the linear filter represents an onset detector with symmetric negative and positive deflection amplitudes. Such a system may arise in the analysis of auditory nerve responses [19] or visual retinal ganglion cells for responses to a sequence of image intensities [5], [20].

A temporal stimulus sequence was created by independently drawing 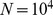 values from a normal distribution, 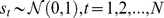, and expanding positive values and compressing negative values. Such positively-skewed distributions often arise for natural images [20]. Figure 4 **B** shows mean across all stimulus dimensions of the modified stimulus distribution. Stimulus examples were created from the stimulus sequence by recasting 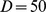 samples preceding the response. Responses were simulated by projecting stimulus examples onto the RF and applying a saturating static nonlinearity with subsequent Poisson spike generation.

Figure 4 **C** shows estimates of the linear filter obtained using the CbRF method, MID analysis and the linear STA estimator (see Methods S1). Both CbRF and MID recover the true linear filter. The correlation between estimated and true RF is 0.99 in both cases. The STA-based estimate, however, suggests that the magnitude of the positive deflection is about half the magnitude of the negative deflections. This is produced by the long tail towards large positive values of the stimulus distribution. Decorrelation of the STA did not enhance performance due to the diagonal stimulus auto-covariance matrix.

#### Robustness to higher-order correlations in the stimulus ensemble

Interactions between higher-order correlations in the stimulus ensemble and nonlinear neural response properties may result in an overestimation of the dimensional support of the RF, even for stimulus ensembles with vanishing second-order correlations [22]. By dimensional support we refer to the dimensions in which the true linear filter is non-zero. Hence, filter estimates obtained with stimuli that contain higher-order correlations may partially reflect stimulus-dependent response properties rather than properties of the true RF. The performance in such scenarios is investigated below for the proposed method and compared to the STA and MID.

**Figure 5:**
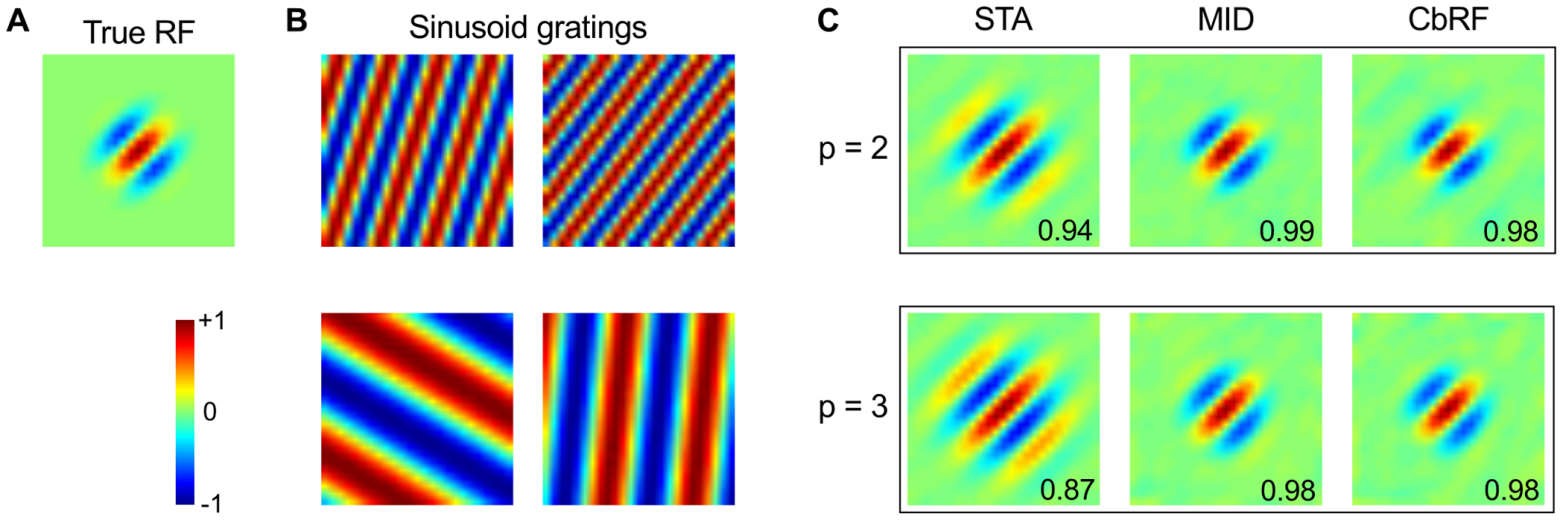
Robustness of RF filter estimation to higher-order correlations in the stimulus ensemble. (**A**) Ground truth linear filter underlying the simulations. (**B**) Examples of sinusoid grating stimuli conveying higher-order correlations. The stimulus ensemble was composed of 80,000 grating stimuli with random orientation and spatial frequency. Second-order correlations were removed by a whitening transformation prior to simulation and analysis. (**C**) Filter estimates obtained with STA, MID and CbRF methods. Quadratic (p  = 2, upper row) and cubic (p  = 3, lower row) nonlinearities were used for LNP model simulations. Overestimation of the RF filter support visible in the STA result is a result of higher-order stimulus correlations, since the stimulus (second-order) auto-covariance matrix was diagonal by construction. Correlation of estimated with true RF filter indicated in lower right corner of each plot.

To separate the effect of higher-order correlations and asymmetric stimulus distributions we used an ensemble of sinusoid gratings, an effective and frequently used stimuli in the visual system, e.g., [2], [7]. The stimulus ensemble consists of 80000 patches of size 25×25 with randomly chosen orientation, spatial modulation frequencies and spatial phase. Figure 5 **B** shows four grating stimulus examples. Second-order correlations were removed by whitening the stimulus ensemble prior to simulation and analysis. The resulting stimulus distribution is spherically symmetric due to the equal-probable positive and negative sinusoidal amplitudes but the stimulus dimensions are not independent due to periodicity of the gratings: non-zero filter values in two dimensions may systematically imply non-zero values in other dimensions ("multi-point interactions'') for the subset of stimulus examples that produce a non-zero response (see Methods S1).

The results are shown in Figure 5 **C**. Already for a quadratic nonlinearity (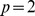; "three-point interactions'', see Methods S1) the STA exhibits systematic and significant overestimation of the dimensional support at multiples of the modulation frequency of the Gabor filter. The effect becomes even more pronounced for a cubic nonlinearity (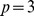; "five-point interactions''). In contrast, CbRF and MID do not show any systematic overestimation of the dimensional support indicating robustness to higher-order correlations in the stimulus ensemble, even in such a distinct example.

#### Receptive field inference from responses to natural stimuli

The stimuli used in real experiments usually contain both second as well as higher-oder correlations and a non-symmetric stimulus distribution 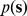. Here, we analyze the capability of the different methods to reconstruct RF parameters from simulated responses to natural stimuli.

As an example, we used human speech taken from the TIMIT speech corpus [44]. To simulate peripheral processing utterances from different speakers have been transformed into octave-like frequency bands using a gammatone filterbank with subsequent log-like compression of the envelope of the filter outputs. The frequency range has been limited to the range in which speech contains a complex harmonic structure, namely between 500 Hz and 4 kHz, and the temporal resolution was set to 2.5 ms.

**Figure 6:**
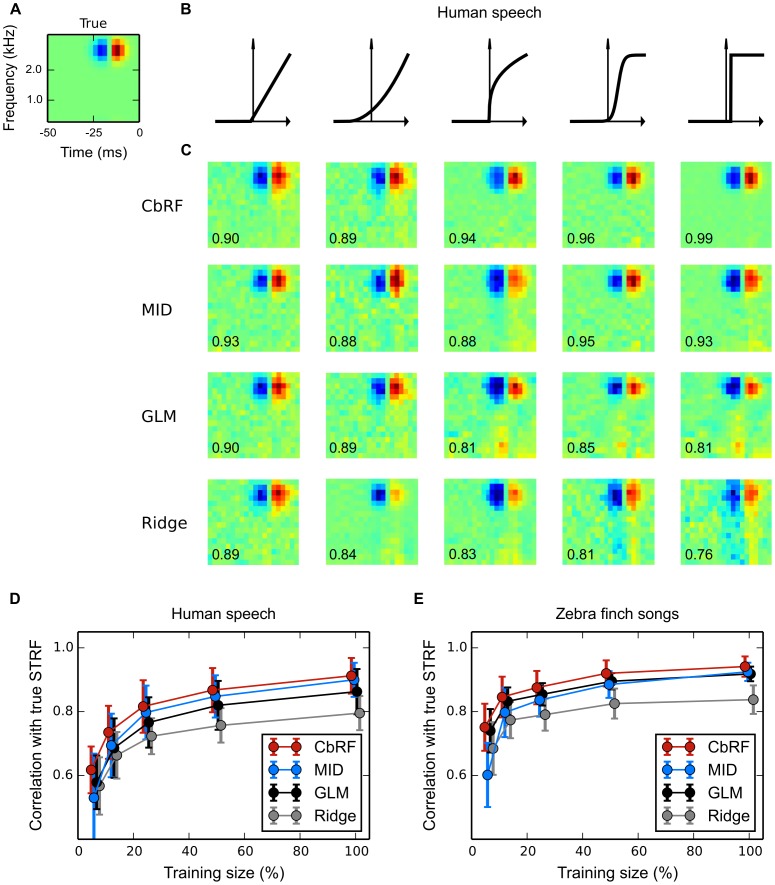
Spectro-temporal receptive field (STRF) estimation from simulated responses to natural stimuli: Robustness to neuronal nonlinearity. (**A**) Ground truth spectro-temporal linear RF filter used in LNP model simulations of spike responses to four minutes of human speech. (**B**) Different static nonlinearities utilized in the LNP model, ranging from linear to step-like, the output of which was used for Poisson process spike train generation. (**C**) Linear RF filter estimates obtained with four estimation methods (rows, explanation cf. Table 1) for each of the nonlinearities in panel **B** (columns). Numbers indicate correlation of estimated with true RF filter. CbRF and MID methods reliably recovered the true linear filters. The GLM shows a bias when the assumed exponential inverse link function deviates from the static nonlinearity used to generate the data, e.g., for the compressive, sigmoid, and threshold nonlinearities. (**D**) Average correlation between true and estimated linear filter for speech stimuli of varying length. An ensemble of model cells was created using different linear filters and different nonlinearities from panel **B** with randomly chosen parameters. Shown are the correlations' mean and standard deviation across 150 model cells for each method. With mean correlation about 0.93 for 100% (four minutes) of the data, CbRF and MID yield higher correlation than GLM and ridge regression. Towards smaller sample sizes, CbRF method performance declines slower than the other methods' including MID's. Bias of the linear ridge regression estimator may be due to the highly non-Gaussian structure of human speech. (**E**) Same experiment as in **D** but with conspecific zebra finch vocalization stimuli of total length three minutes. CbRF method resulted in highest mean correlation for all stimuli lengths. GLM and MID method showed similar performance for long stimuli with GLM declining less towards smaller sample sizes below 50%. The somewhat higher mean correlation values observed for ridge regression in comparison to panel **D** may be attributed to the fact that the zebra finch vocalizations were less non-Gaussian than human speech.

Responses were simulated using a narrow-band onset spectro-temporal receptive field (STRF) (Figure 6 **A**), a pattern that has been found throughout different stages of the auditory system of mammals [10], [15], [45]. The output of the linear stage was transformed into a spike rate using different nonlinearities, ranging from linear to step-like (cf. Figure 6 **B**). Spikes were generated from the spike rate by an inhomogeneous Poisson process. We strove to achieve a realistic average spike rate between 0.02 and 0.1 spikes per sample for all nonlinearities.

Figure 6 **C** shows linear filter estimates for the onset filter produced by ridge regression, a GLM with Poisson distributed noise, MID, and the CbRF method (see Methods S1 for details on ridge regression, the GLM and MID). For the half-wave rectified linear and quadratic nonlinearities, GLM, MID and CbRF perform almost identically, obtaining a correlation of approximately 0.9 with the true filter. With increasing degree of nonlinearity the performance of the GLM decreases. This is likely a result of a mismatch in the assumed nonlinearity, which is exponential for the GLM, and the nonlinearity used to produce the spike trains. MID and CbRF were able to reliably recover the true linear filters. Due to the strong non-Gaussian structure of speech the linear ridge estimator shows a strong bias, in particular for nonlinear model cells.

We also tested dependence of the different methods on data set size. Therefore we simulated responses with varying number of samples using the different nonlinearities and estimated the linear filter using the different methods. To obtain a diverse ensemble of responses we also used different linear filters, e.g., frequency-shifted versions of the above onset filter and Gabor-like filters of different orientations. We further randomly varied the parameters of the different nonlinearities, e.g., the exponent of the compressive nonlinearity or the spiking threshold of the threshold nonlinearity, resulting in 150 distinct model cells for each sample size.

The results are shown in Figure 6 **D**. For 100% of the data (corresponding to 4 minutes of speech) MID and CbRF show comparable performance. With decreasing sample size the CbRF method yields noticeable higher average correlations with the true linear filter than MID. The performance of the GLM is below the CbRF by about 5%, a result of bias in GLM-based estimates for static nonlinearities not matching the GLM's inverse link function as described above. Thus, across all model cells the CbRF method is more robust to different nonlinearities than the GLM while being less sensitive to small sample sizes than MID.

Figure 6 **E** shows the results for the same experiment but with zebra finch vocalizations as stimulus instead of human speech. The zebra finch vocalizations were provided by the Theunissen lab through the CRCNS database [46], and have previously been used as stimuli in neurophysiological experiments [47], [48]. Similar to the speech experiment, the CbRF method yields considerable higher mean correlation values than MID with decreasing sample sizes. However, the GLM shows improved performance compared to human speech, outperforming MID for sample sizes below 50%. This may be a result of the less non-Gaussian structure of zebra finch songs compared to human speech. This also becomes apparent for the ridge method that yields higher mean correlation values. However, across different model cells and stimulus classes, the CbRF method provided the best performance, in particular for small sample sizes.

### Receptive Field Estimation from Experimental Responses

The method was then tested using data from single-unit recordings in the inferior colliculus (IC) of anesthetized Mongolian gerbils. Stimuli for STRF estimation consisted of consecutive blocks of frequency-modulated (FM) tone complexes (see Materials and Methods). FM tones have been shown to constitute important auditory features, e.g., phoneme transitions in human speech or conspecific vocalizations, manifesting in the sensitivity of the auditory system to spectro-temporal transients [10], [49], [50], and can be considered as partial analogue to visual spatiotemporal edges [51]. Furthermore, temporal amplitude transients induced by the block structure have shown to be an essential feature of the auditory system [52], [53]. Hence the used FM complex stimuli might bear advantages for investigating neuronal processing of specific aspects of natural sounds. We also probed IC units with FM sweeps that continuously start in time. For details on experimental procedures and stimulus generation see Materials and Methods.

**Figure 7:**
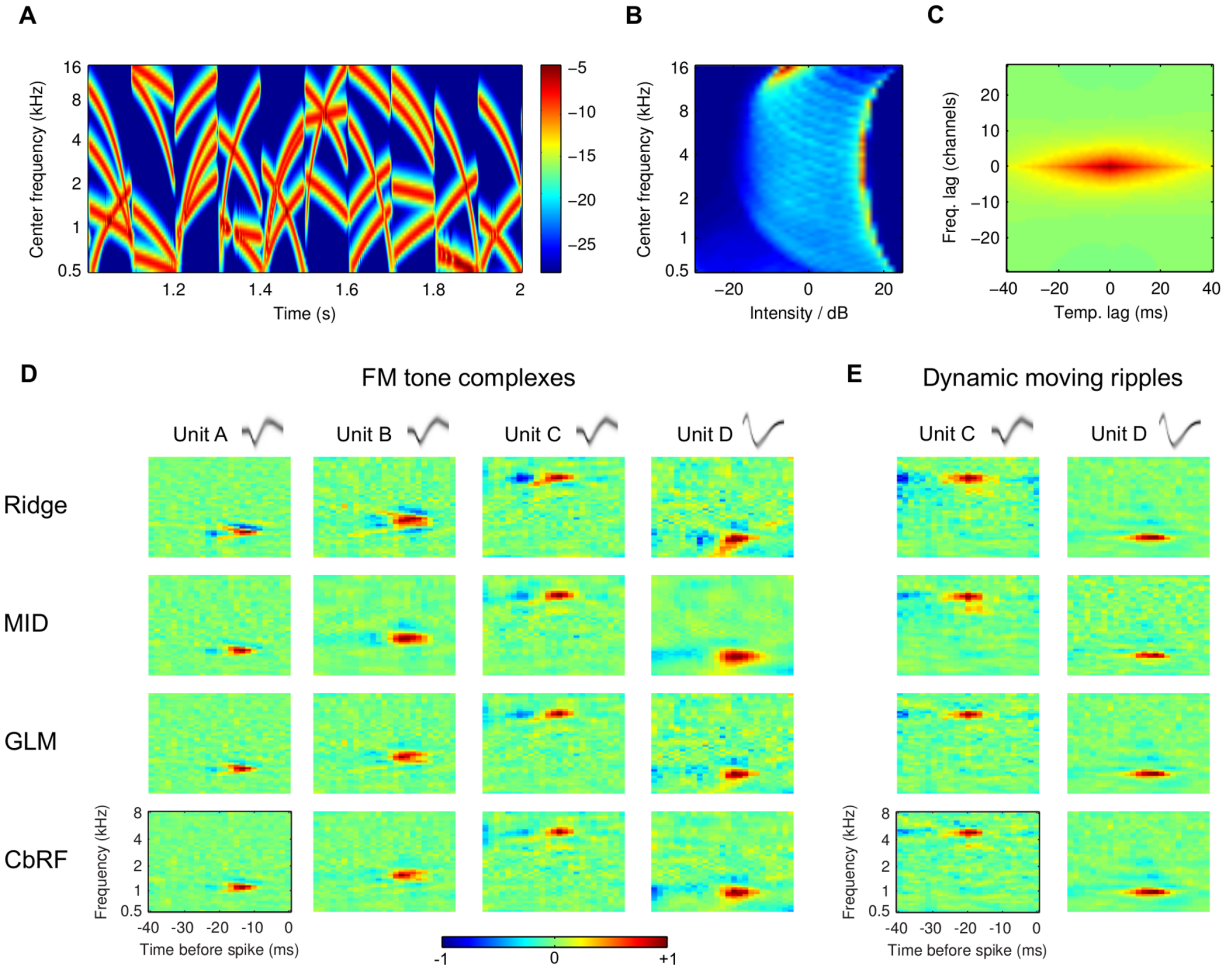
STRF estimation from gerbil inferior colliculus (IC) responses to frequency-modulated (FM) sweep complex stimuli. (**A**) Example segment of block-design FM tone complex with length 1 s. Amplitude scaling in decibel (dB), dynamic range limited to 25 dB below maximum for visualization. (**B**) Stimulus amplitude histogram, shown for each spectral band after centering; red (blue) indicate high (low) probability, respectively. (**C**) Normalized spectro-temporal auto-correlation function of stimulus ensemble. (**D**) STRFs estimated from recorded responses of four gerbil IC units (columns) with four inference methods (rows, explanation cf. Table 1). All units had best frequency below 8 kHz and analysis was restricted to the range 0.5 kHz to 8 kHz. The spike waveform density function of each unit is shown on top of each column, verifying single-unit activity [66]. Spectro-temporally transient ("diagonal'') patterns that are exhibited in the ridge regression-based estimates (top row) lack confirmation in the MID-, GLM-, and CbRF-derived STRF estimates (lower three rows). Thus, we hypothesize that these are an artefactual result originating from higher-order correlations and distribution asymmetries within the stimulus ensemble which the ridge regression method is not robust to. In general, MID, GLM, and CbRF produce very similar STRF estimates, with the latter two methods revealing a slightly finer tuning in some cases. (**E**) Validation experiment with dynamic moving ripple (DMR) stimuli responses recorded from two identical units (units C and D) as shown in experiment panel **D**. Spectro-temporal transients absent in all methods' STRF estimates, presumably due the absence of higher-order correlations in the DMR stimuli and consistent with the explanation of panel **D** results.

Figure 7 **A** shows a 1 s segment of an FM complex stimulus spectrogram. The block length is 0.1 s and each block contains four sweeps with randomly chosen starting and ending frequencies. The stimulus distribution is shown in Figure 7 **B**. Stimulus examples were sampled from the stimulus spectrogram by recasting spectro-temporal patches preceding the response in a 40 ms time window as vectors. Thus, the statistics of the stimulus ensemble is well approximated by the distribution of samples in each frequency channel, which is clearly non-Gaussian in this case (mean skewness 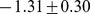). As indicated in Figure 7 **C**, second-order correlations in the stimulus ensemble are most pronounced in temporal direction spanning the whole patch size. This is a result of the high temporal resolution (2 ms) of the filter bank corresponding to the bin width of the spike trains. All units had a best frequency below 6 kHz. Therefore, we restricted the analysis to the range 0.5 kHz to 8 kHz resulting in 900-dimensional stimulus vectors.

Example STRFs for four units estimated using ridge regression, MID, GLM, and the CbRF method are shown Figure 7 **D**. The ridge estimator shows both diagonal ("sweep like'') structures and a stronger negative deflection compared to the other methods. Taking into account the results for simulated responses, the diagonal structures may be a result of higher-order correlations, whereas the increased negative deflection is likely caused by the long tail of the stimulus distribution towards negative values (see Materials and Methods).

Compared to MID both GLM and CbRF reveal slightly finer spectro-temporal tuning in some cases. In general, the three methods reveal almost the same STRF structure. There was a high correlation between STRFs derived using CbRF and the GLM (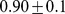 for the 38 IC units probed with FM sweep complexes arranged in blocks; 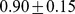 for the 38 IC units probed with continuously starting FM sweeps). The mean correlation between STRFs for MID and CbRF was only slightly lower (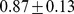 for the 38 IC units probed with FM sweep complexes arranged in blocks; 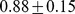 for the 38 IC units probed with randomly starting FM sweep stimuli). For comparison, the mean correlation between STRFs estimated using CbRF and ridge regression was 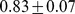 and 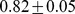 for the block-like and continuously starting stimulus ensembles, respectively.

Units C and D in Figure 7 **D** were also probed with dynamic moving ripple (DMR) stimuli as described in [54]. DMR have successfully been used in the IC in cats and allow STRF estimation using linear estimators like ridge regression [55]. The DMR-based STRFs are shown in Figure 7 **E**. The absence of diagonal and strong inhibitory structures suggests that MID, GLM and the CbRF method produced robust estimates of spectro-temporal integration mechanisms for the units.

#### Population Analysis

For a quantitative evaluation the data is split into two different parts: one part for training the model (80%) and one part to evaluate the model on unseen data (20%). This is done for different parts of the data in a 5-fold cross-validation scheme. The regularization parameter is found by cross-validation on the training data. We used mutual information (MI) between stimulus and response for evaluation of the STRF estimates (see Methods S1). MI is a model-independent measure and does not depend on the scaling of the STRFs, which is inherently different for all methods. Marginal and spike-conditional probability densities were estimated using histograms.

We also included the "plain'' STA and the normalized reverse correlation (NRC, [11]) method, a variant of the STA that uses a different regularization scheme than ridge regression (see Methods S1). The NRC estimator has been used as a reference a in number of studies, e.g., [8], [27], [56], [57], and has been included to allow a better comparison across studies.

**Figure 8:**
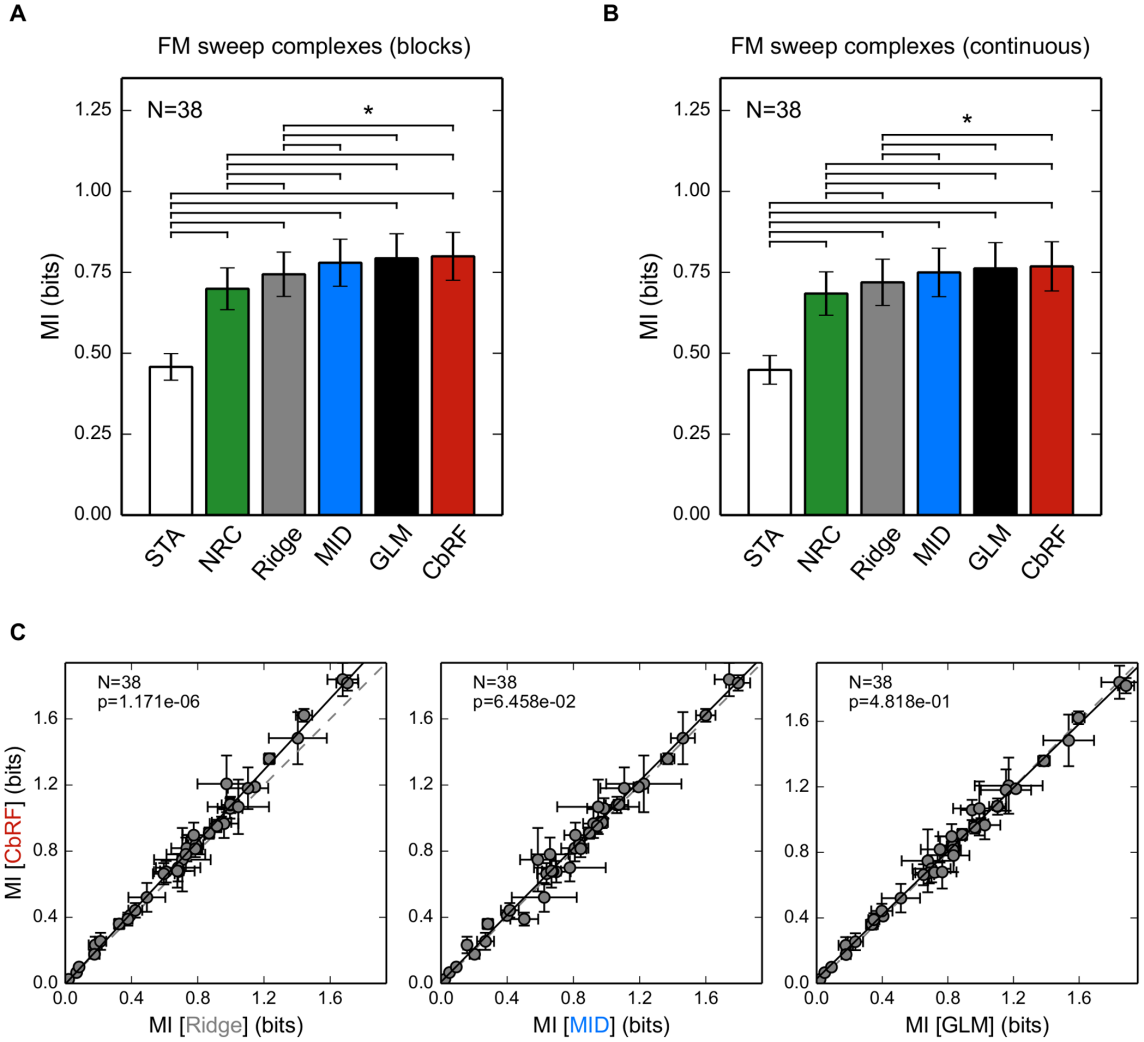
Population analysis of STRF estimation for gerbil IC units using FM sweep complex stimuli in block–design and continuous–onset–design. (**A**) Predictive power for the different methods in terms of cross-validated mutual information (MI) between stimulus and response, showing mean and standard error. CbRF, MID and GLM perform almost identically with no significant difference between the methods. Linear estimators (STA, NRC, ridge regression) show significantly lower predictive power. 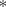 denotes statistical significance (paired Wilcoxon test; 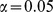). (**B**) Same experiment as panel **A**, but for 38 IC responses to continuously starting FM sweep complexes recorded in a separate neural subpopulation. (**C**) Predictive power of the CbRF method for single units compared to ridge regression, MID and GLM. Shown are mean and standard deviation across five cross-validation folds for the 38 IC units in panel **A**.

Figure 8 **A** summarizes mean and standard error for cross-validated MI values for the different methods for the neural subpopulation probed with FM sweep complexes arranged in blocks. Across all 38 units, MID, GLM, and the CbRF method show a significantly higher predictive power than the linear estimators, namely STA, NRC, and ridge regression (paired Wilcoxon test; 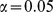). GLM and CbRF yield slightly higher but not significant mean predicted MI values than MID (paired Wilcoxon test; 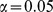). Example scatter plots comparing cross-validated MI for the CbRF method to ridge regression, MID, and the GLM for the 38 units are shown in Figure 8 **C**. Detailed comparisons of cross-validated MI values for the other methods are shown in Figure S1. All 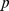-values have been adjusted using the Holm–Bonferroni method.

There is also a significant difference in cross-validated MI between the linear estimators. Ridge regression shows significantly higher predictive power than the NRC method (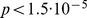; paired Wilcoxon test) and the STA (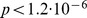; paired Wilcoxon test). Whereas the STA is biased due to second- and higher-order correlations, the only different between NRC and ridge regression is the regularization method (see Methods S1 and Discussion). The results for the second ensemble of IC responses to FM sweep stimuli with continuously starting sweeps shown in Figure 8 **B** confirm these findings. Thus, across a large ensemble of IC responses to non-Gaussian stimulus ensembles the CbRF method allows reliable estimation of STRF parameters.

#### Convergence Properties

The time we are able to record from one or more units usually restricts the available dataset to some noisy observations. In other situations, we may want to study neural effects, e.g., adaptation to stimulus statistics, that may take place on time scales smaller than the time a method needs to converge [8]. In both situations, the goal is to achieve accurate estimates with a possibly small amount of data.

To test convergence properties on neural recordings, we estimated STRFs using 10%, 25%, 50%, 75%, and 100% of the experimental IC data. To mimic a real recording situation we always started from the beginning of the recording. Since the "ground truth'' STRF is not known and the employed MI measure depends on the total information in the response for each unit and may become highly biased for small sample sizes, we used the correlation between partial STRF estimates and the STRF estimated using all data. Hence, the obtained convergence curves represent relative convergence and the population MI results have to be taken into account for comparison of the overall performance.

**Figure 9:**
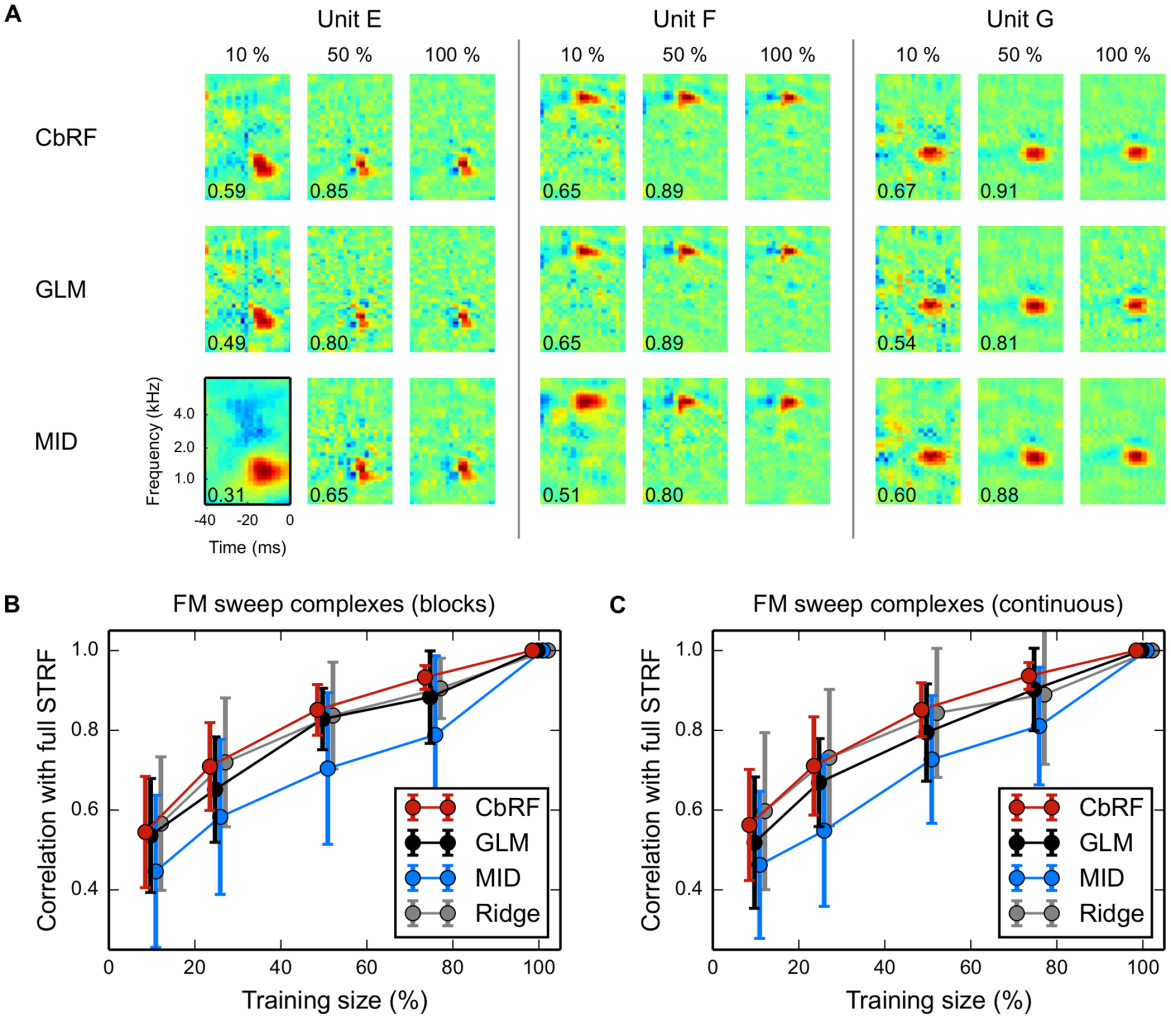
Convergence properties. STRFs have been estimated using a subset of the data and compared to the full data estimates as described in the text. (**A**) Example STRFs for three units estimated using CbRF, GLM, and MID using 10%, 50%, and 100% of the data, respectively. Numbers in each STRF plot indicate correlation with the corresponding full (100%) estimate. (**B**) Relative convergence curves showing mean and standard deviation across 38 IC units for the block–design FM sweep complex stimuli for CbRF, GLM, MID and ridge regression. The CbRF method shows an average correlation of 0.9 (0.8) with the full STRF estimate for about 60% (35%) of the data. MID requires more than 87% (76%) to reach the same correlation. Performance of the GLM is between CbRF and MID. Across all experiments, CbRF has consistently lower standard deviations than MID, GLM and ridge estimation. Note that by contruction all curves reach correlation one with standard deviation 0 at training size 100%. Bars were shifted horizontally for visualization purposes (with GLM at correct horizontal locations). (**C**) Same experiment as in **B**, but for 38 IC responses to continuously starting FM sweep complexes.

Example STRFs for three units estimated using CbRF, GLM, and MID are shown Figure 9 **A**. In all cases, the CbRF method produces estimates very close to the final estimates using about 50% of the data as indicated by the high correlation between partial and full estimates. To quantify this, we calculated the correlation for all data sets. Figure 9 **B** and Figure 9 **C** display mean and standard deviation for the IC responses to FM sweeps in blocks and continuously starting FM sweeps, respectively. For all conditions, the CbRF approach yields higher mean correlation values than MID reaching a similarity of 90% with its final estimate with approximately 60% of the data. MID requires on average more than 90% of the data to reach the same mean correlation value. The variability of the CbRF-derived estimates indicated by the standard deviation is much smaller than for the other methods. For comparison, the GLM has also been tested showing intermediate performance compared to CbRF and MID.

## Discussion

We have described a novel classification-based receptive field (CbRF) estimation approach to infer receptive field (RF) parameters from binary spike/non-spike predictions in a high-dimensional stimulus space. In this interpretation, the problem of RF estimation corresponds to finding the linear classification rule that optimally separates stimulus examples that elicited a spike from those that do not. The underlying threshold model includes an essential characteristic of the spike generation process and a simple model with Gaussian noise is sufficient to explain a wide range of nonlinearities, ranging from almost linear to step-like [18]. The assumption of a monotonic nonlinearity restricts the model to cells for which the spike-triggered average (STA) is non-zero [17]. However, such cells occur at many stages of the sensory system, e.g., in the IC of gerbils and cats [45], [55], in the A1 of ferrets [13], [16], and in V1 in cats [8], [57].

We have also presented an algorithm based on a linear large-margin classifier with the goal of minimizing the empirical risk of misclassifying stimulus examples. The objective function is convex and solutions of the resulting optimization problem can be found efficiently. Although the underlying loss function aims at Bayes optimal decision rule [30], [31], this approach learns RF parameters without specifying any density function and, therefore, differs from maximum likelihood estimation of model parameters [9], [21], [25], [27]. On the other hand, recent analysis has shown that the support vector machine (SVM), a problem highly related to the proposed approach, may be viewed as a maximum likelihood estimate of a class of probabilistic models [36]. Furthermore, in case the loss function can be expressed as the negative log-likelihood risk minimization and likelihood maximization are numerical identical, i.e. penalized logistic regression.

To elucidate the importance of class priors for the CbRF approach we also estimated STRFs from the IC recordings without weighting errors by the respective inverse class prior (cf. Figure S2). Without weighting, STRF estimates reveal stronger negative deflections and slight diagonal structures as for the ridge modification of the STA. Moreover, comparing cross-validated mutual information (MI) values of weighted and unweighted estimators reveals that weighting significantly increases predictive power (cf. Figure S2; weighted vs unweighted: 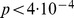; paired Wilcoxon test). These results indicate that this approach does not work with a standard classifier and validate theoretical considerations regarding highly unbalanced binary problems [33], [36].

We have shown that the CbRF estimator is robust against second-order and higher-order correlations in the stimulus ensemble, thus alleviating the Gaussian assumptions of the STA method. Even though higher-order correlations contribute only a small fraction to the quantitative entropy measure of information explained in natural signals (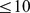 %, [58]), RF estimates obtained using a linear STA estimator may suggest qualitative difference in response characteristics [8], [22]. E.g., the diagonal structures in STA-based STRF estimates in Figure 0, which are presumably induced by the stimulus ensemble composed of frequency-modulated tone complexes and do not represent neural response properties. This is confirmed by the absence of such diagonal structures for DMR stimuli that only contain correlations up to and including second order, making them suitable for STA-based response characterization [54], [55], [59]. However, frequency modulations are prominent features of natural signals [49], [60]. Thus, a robust description of stimulus-response properties for such stimulus ensembles, particularly with regard to natural stimuli, is fundamental for neural response characterization.

### Differences between the Different Estimation Methods

All estimation methods apart from MID and NRC can be formulated as 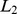-norm regularized optimization problem (cf. Eq. (2) in Materials and Methods). Thus, the only computational differences are the employed cost functions and the optimization strategies. Table 1 summarizes cost functions, regularizers, optimization algorithms, and model selection criteria for the different methods.

**Table 1:** Summary of estimation methods with associated cost function, regularizer and optimization technique.

Method	Cost	Regularizer	Criterion	Optimizer
STA	Least squares	None	n/a	Closed form
Ridge	Least squares	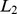	MI	Closed form
NRC	Least squares	Truncated SVD of cov. matrix	MI	Closed form
MID	MI	Early stopping	MI	SA+gradient ascend
GLM	Poisson log-likelihood	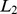	MI	Trust region Newton CG
CbRF	Squared hinge loss	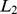	AUC	Trust region Newton CG

Ridge, GLM and CbRF methods put a Gaussian prior on the filter coefficients, implemented by the 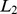-norm penalty in the objective function. The squared hinge loss of the CbRF method corresponds to the least squares loss with truncated negative part. Cross-validation on training data is used for all methods to determine the optimal regularization hyperparameter or the termination of early stopping for MID. STA: spike triggered average [19]. Ridge: ridge regression [67]. NRC: normalized reverse correlation [11]. CbRF: proposed classification-based receptive field estimation. GLM: generalized linear model [25], [26]. MID: maximally informative dimensions [18]. MI: mutual information between stimulus and response. SVD: singular value decomposition. CG: conjugate gradient. AUC: area under receiver operating characterisitc curve. SA: simulated annealing.

STA, ridge regression and NRC are linear estimators that seek to minimize the mean-squared error between model predictions and neural data (for details see Methods S1). The difference between the latter two methods is the employed regularization method. NRC performs linear regression in a subspace spanned by the eigenvectors of the covariance matrix whereas ridge regression assumes a multivariate Gaussian distribution of the linear filter parameters. As discussed in [27] NRC tends to remove high frequency components for signals with low-pass characteristics, e.g., natural stimuli, resulting in broadened STRF estimates. We also found this effect in the NRC-based STRF estimates. Ridge regression revealed STRF estimates with finer tuning and thus higher predictive power. For both methods we also compared closed-form regression solution and different iterative gradient-based optimization techniques and found no significant difference in predictive power (paired Wilcoxon test; 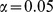). Furthermore, replacing the MI-based optimization criterion to find the regularization hyperparameter by area under ROC curve (AUC) or mean squared error did not increase predictive power (paired Wilcoxon test; 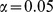).

In comparison with linear estimators, the CbRF method reliably recovered the true linear filter from simulated responses to natural stimuli and revealed higher predictive power on IC data. Both CbRF and ridge regression use the same Gaussian prior on the linear filter coefficients (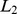-norm regularization). Thus, the only quantitative difference is the employed loss function. Considering that the CbRF's loss function corresponds to the least squares loss of ridge regression with truncated negative part the improved performance of the CbRF method may seem surprising. However, the underlying empirical risk minimization principle is different from the least squares approach, which assumes a Gaussian distribution of the data. Empirical risk minimization in the form of a large-margin classifier does not make any stimulus distribution-related assumptions, which seems to be crucial for reliable estimation of RF parameters.

The GLM fits the data to a Poisson distribution and relates the linear part to the spike response via an exponential nonlinearity. The exponential is the canonical inverse link function for the Poisson distribution and other choices are possible. However, as demonstrated in [27], the specific type of link function seems to have minor influence on the predictive power of the GLM. We also tested a half-wave rectified linear inverse link function on a subset of both IC data sets and did not find a significant difference in predictive power (paired Wilcoxon test; 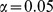). The influence of spike interactions in the form of a post-spike filter were tested on a subset of the IC data. However, there was no increase in predictive power or convergence speed and in some cases we even found a decrease in performance.

The CbRF method revealed higher robustness to different nonlinearities than the GLM on simulated data. To explore the comparable predictive power of GLM and CbRF on neural recordings we analyzed the neural nonlinearities in the IC data. Both approaches reveal largely expansive (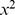-like) nonlinearities (cf. Figure S3). The average correlation between nonlinearities inferred from STRFs estimated using the CbRF method and the GLM is 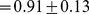. Thus, the equivalent performance of GLM and CbRF on the IC recordings seems to be a result of nonlinearities that may be well approximated by the GLM's exponential nonlinearity.

We found a significant influence of the optimization algorithm on the performance of both CbRF and GLM, in particular for highly correlated stimulus features. Amongst all tested gradient descend algorithms, e.g., conjugate gradient (CG), truncated Newton CG, and the Broyden–Fletcher–Goldfarb–Shanno algorithm, the employed trust region Newton CG algorithm showed the best performance on both simulated data and IC recordings. Compared to the GLM the CbRF allows more efficient computation of the model parameters. As a result of the CbRF's truncated least squares loss the gradient needs only to be updated for the subset of misclassified stimulus examples, allowing the combination of low computational cost and fast convergence of the employed trust region optimization algorithm [40]. We found a speedup of about 2–10 for the CbRF method compared to the GLM.

Both CbRF and MID effectively aim at the model that minimizes the overlap between the distributions of spike-conditional and non-spike-conditional projections onto the linear filter (see Materials and Methods). The high correlation between STRFs produced by the two approaches in the large-data regime indicates that there is not only a conceptual but also a quantitative similarity. However, the underlying empirical risk minimization principle is different from maximization of MI between stimulus and response. This becomes apparent for small sample sizes, a scenario in which the concept of class separation makes it sufficient to "collect'' some stimulus examples close to the separating hyperplane to obtain an approximate solution to the problem [34], [35], [61]. This also enhances performance in case the number of spikes is rather small (cf. Figure S4). In contrast, information-theoretic approaches use histogram-based estimation of probability distributions, which is prone to be biased in some data regimes even if the correct estimator is used [62]. The AUC metric implicitly optimized by the CbRF has been shown to significantly reduce bias compared to MI for rather small sample sizes while being highly correlated with MI in the large-data regime [63].

### Limitations of the CbRF Method

In comparison to the proposed CbRF method, estimators like the spike-triggered covariance (STC) are much more general allowing RF estimation for cells with symmetric nonlinearity (for which the STA is zero) and extension to several linear filters spanning the relevant subspace of a neuron for Gaussian stimuli [3], [17]. Information-based approaches extend this concept to non-Gaussian stimulus ensembles, e.g., natural stimuli [18], [23], [58], [64]. However, similar to the generalized quadratic model [65] the CbRF may be extended to several filters by augmenting a quadratic component. The resulting estimator seeks the separating hyperplane in an second-order polynomial space. Such an approach may be implemented efficiently in terms of a polynomial kernel [35], [61]. Thus, the CbRF method may even allow characterization of the relevant subspace spanned by several linear filters. In the current version, the proposed CbRF approach represents an alternative technique to infer single filter parameters from responses to non-Gaussian stimulus ensembles that may be beneficial in case data is rare or if the number of observable spikes is small.

## Supporting Information

Figure S1 **Cross-validated mutual information for 38 IC units.** Scatter plots showing mean and standard deviation of 5-fold cross-validated MI for the FM tone complexes with block structure for the different methods.
(TIFF)

Figure S2 **Classification-based STRF estimation with and without class priors.** (**A**) Example STRFs for two units with and without weighting of misclassification errors by inverse class priors. STRFs estimated using the unweighted version show stronger negative deflections and diagonal-like structures similar to ridge regression. (**B**) Predictive power of classification-based STRF estimates in terms of cross-validated MI with and without weighting of errors. STRFs estimated using the weighted version result in significantly higher MI predictions (paired Wilcoxon test).
(TIFF)

Figure S3
**Neural nonlinearities inferred from the IC recordings.** Neural nonlinearities estimated from 38 IC responses to FM tone complexes arranged in blocks. The nonlinearities were constructed by filtering the stimulus ensemble with the STRF, 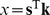, and forming the ratio 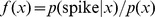. 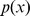 and 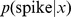 were estimated using histograms (11 bins). (**A**) Nonlinearities constructed from STRFs estimated using the CbRF method. (**B**) Nonlinearities constructed from STRFs estimated using the GLM. In both cases, most nonlinearities reveal an expansive shape that may be well fitted using the GLM's exponential inverse link function. The average correlation between the 38 nonlinearities for CbRF and GLM is 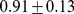.(TIFF)

Figure S4 **Relation between correlation with full STRF and number of spikes.** For each IC unit STRFs were estimated using 10%, 25%, 50%, and 100% of the data. Each dot represents the relation between the number of spikes used for STRF estimation and the correlation with the STRF estimated using 100% of the data. (**A**) Results for 38 IC responses to FM tones arranged in blocks. (**B**) Results for 38 IC responses to FM tones continuously starting in time. The number of spikes was constant across all methods. Thus, any differences in correlations result from the performance of the different methods. The CbRF method reveals noticeable higher correlation values than MID, in particular for small numbers of spikes.
(TIFF)

Methods S1 MID, GLM and STA methods.
(PDF)
