# Supplementary figures and images for: Discriminative Learning of Receptive Fields from Responses to Non-Gaussian Stimulus Ensembles
Source: PLoS One. 2014 Apr 3;9(4):e93062. doi: 10.1371/journal.pone.0093062 (PMC3974709; doi:10.1371/journal.pone.0093062)

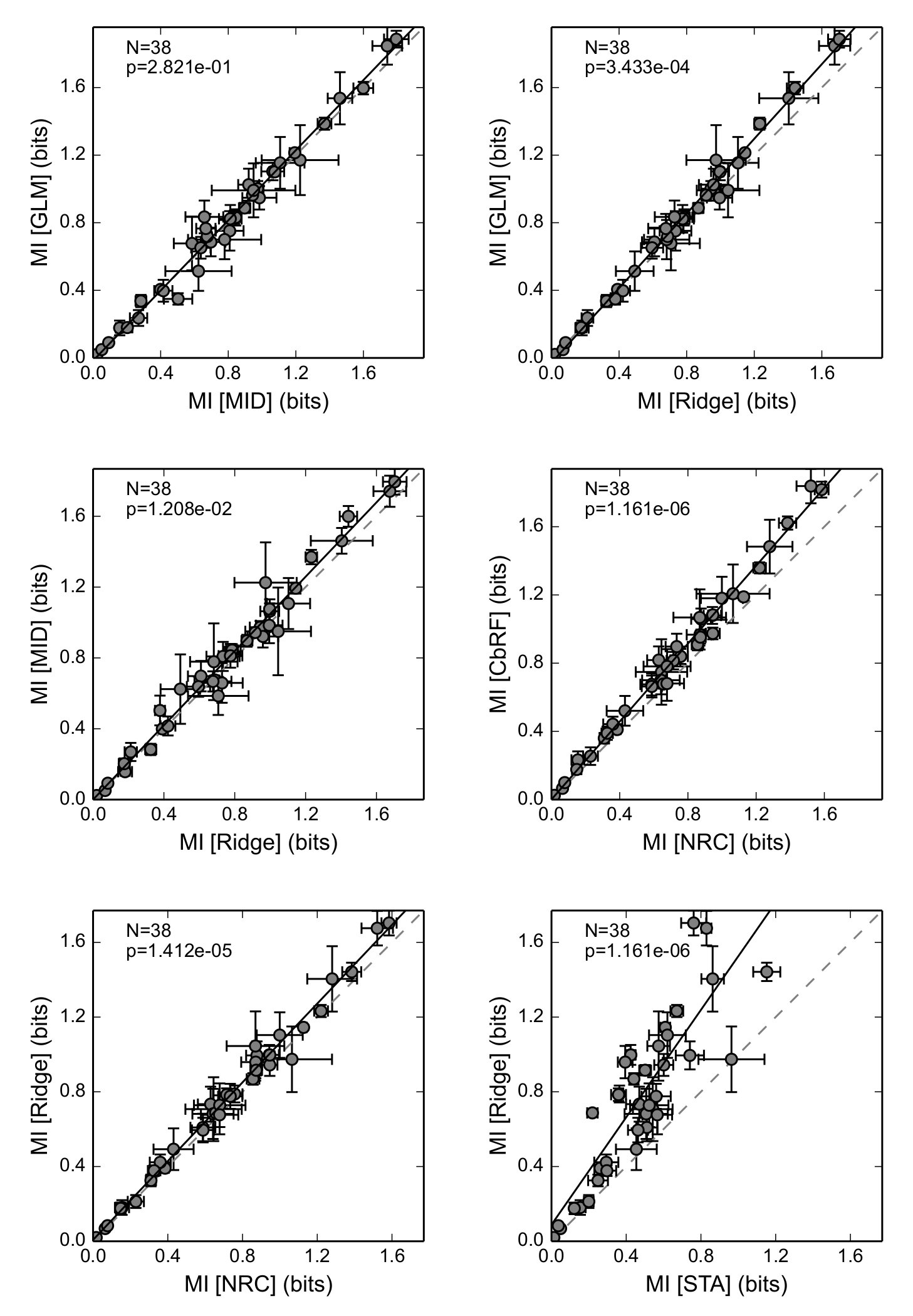

Supplement: Figure S1 — Cross-validated mutual information for 38 IC units. Scatter plots showing mean and standard deviation of 5-fold cross-validated MI for the FM tone complexes with block structure for the different methods. (TIFF) [file pone.0093062.s001.tiff]

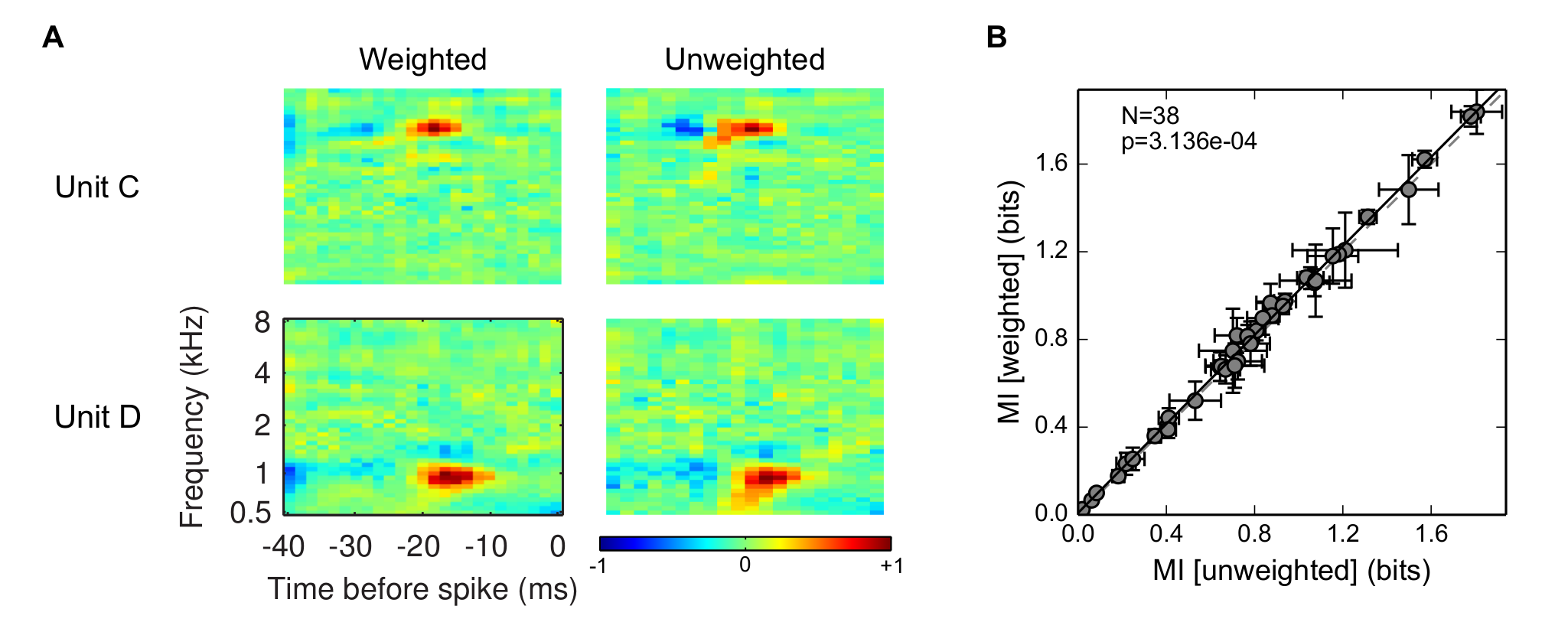

Supplement: Figure S2 — Classification-based STRF estimation with and without class priors. (A) Example STRFs for two units with and without weighting of misclassification errors by inverse class priors. STRFs estimated using the unweighted version show stronger negative deflections and diagonal-like structures similar to ridge regression. (B) Predictive power of classification-based STRF estimates in terms of cross-validated MI with and without weighting of errors. STRFs estimated using the weighted version result in significantly higher MI predictions (paired Wilcoxon test). (TIFF) [file pone.0093062.s002.tiff]

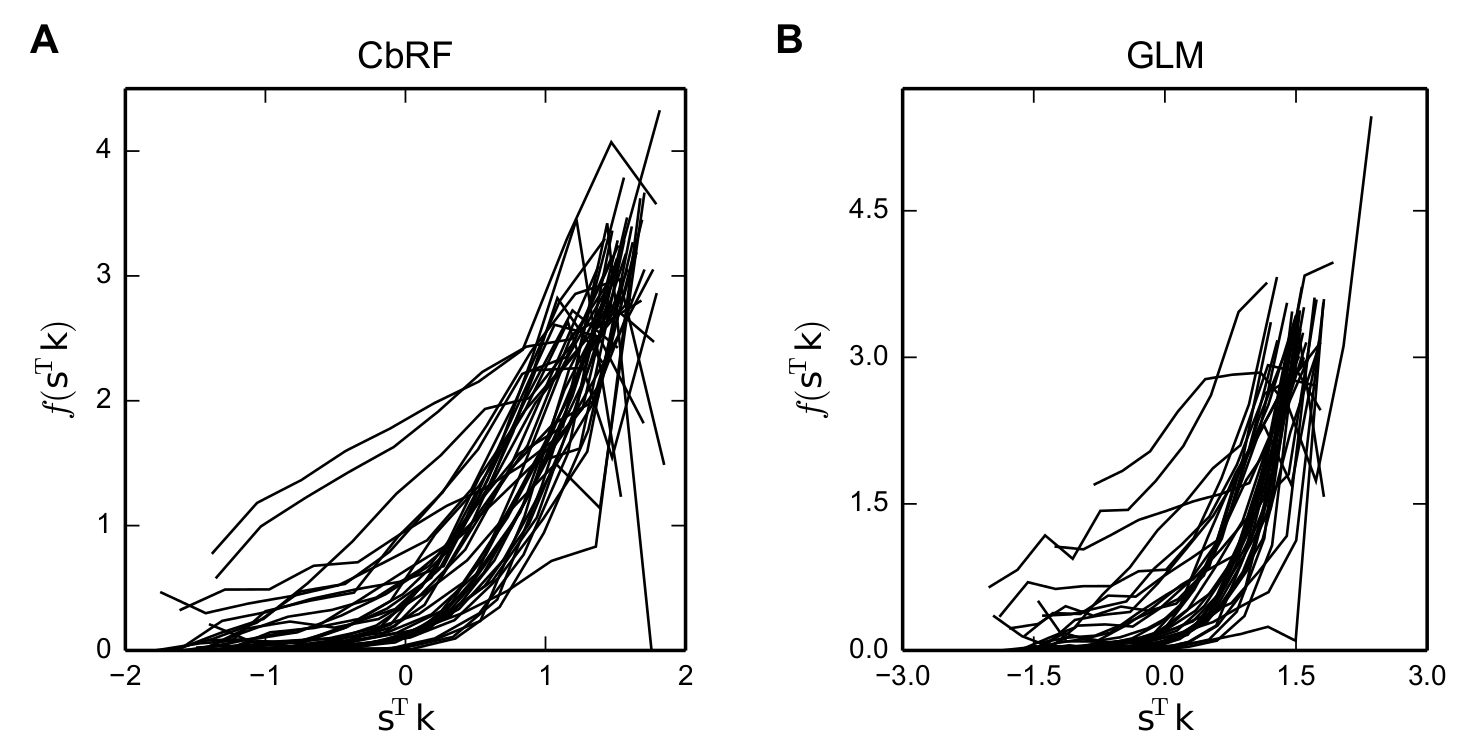

Supplement: Figure S3 — Neural nonlinearities inferred from the IC recordings. Neural nonlinearities estimated from 38 IC responses to FM tone complexes arranged in blocks. The nonlinearities were constructed by filtering the stimulus ensemble with the STRF, , and forming the ratio . and were estimated using histograms (11 bins). (A) Nonlinearities constructed from STRFs estimated using the CbRF method. (B) Nonlinearities constructed from STRFs estimated using the GLM. In both cases, most nonlinearities reveal an expansive shape that may be well fitted using the GLM's exponential inverse link function. The average correlation between the 38 nonlinearities for CbRF and GLM is . (TIFF) [file pone.0093062.s003.tiff]

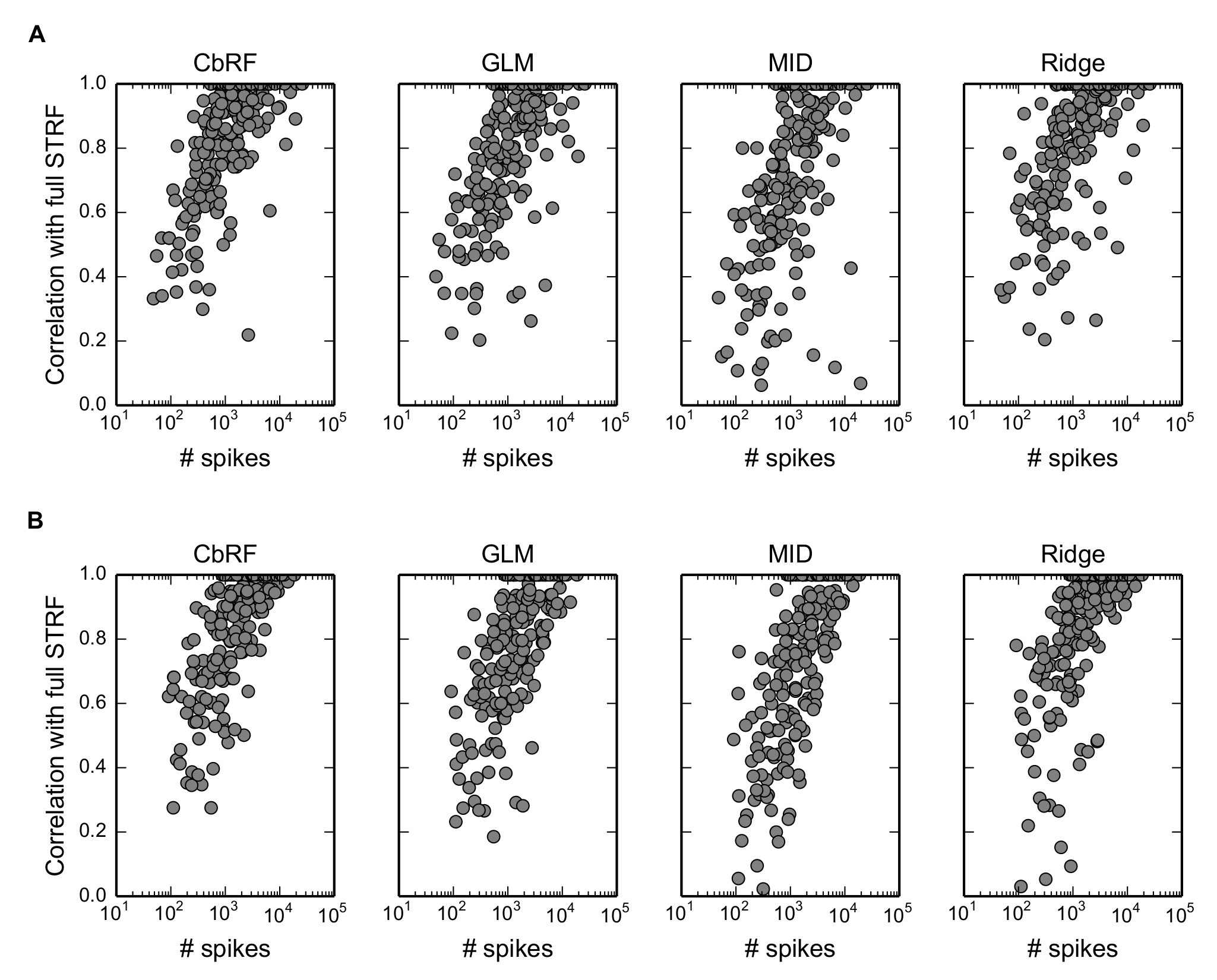

Supplement: Figure S4 — Relation between correlation with full STRF and number of spikes. For each IC unit STRFs were estimated using 10%, 25%, 50%, and 100% of the data. Each dot represents the relation between the number of spikes used for STRF estimation and the correlation with the STRF estimated using 100% of the data. (A) Results for 38 IC responses to FM tones arranged in blocks. (B) Results for 38 IC responses to FM tones continuously starting in time. The number of spikes was constant across all methods. Thus, any differences in correlations result from the performance of the different methods. The CbRF method reveals noticeable higher correlation values than MID, in particular for small numbers of spikes. (TIFF) [file pone.0093062.s004.tiff]
