# Supplementary material for: Discriminative Learning of Receptive Fields from Responses to Non-Gaussian Stimulus Ensembles
Source: PLoS One. 2014 Apr 3;9(4):e93062. doi: 10.1371/journal.pone.0093062 (PMC3974709; doi:10.1371/journal.pone.0093062)
Supplement: Methods S1 — MID, GLM and STA methods. (PDF) [file pone.0093062.s005.pdf]

Supporting Information to “Discriminative Learning of Receptive Fields from Responses to non-Gaussian Stimulus Ensembles”

Arne F. Meyer[1,*], Jan-Philipp Diepenbrock[2], Max F.K. Happel[2,3], Frank W. Ohl[2,3], Jörn Anemüller[1]
1 Department of Medical Physics and Acoustics and Cluster of Excellence “Hearing4all”, University of Oldenburg, Oldenburg, Germany
2 Department of Systems Physiology of Learning, Leibniz Institute for Neurobiology, Magdeburg, Germany
3 Department of Neuroprosthetics, Institute of Biology, Otto-von-Guericke University, Magdeburg, Germany
∗ E-mail: arne.f.meyer@uni-oldenburg.de

Information-based Receptive Field Estimation

Information-theoretic RF estimation seeks to find one or more linear filters of a neuron irrespective of employed stimulus ensemble and neural nonlinearity [1,2]. The goal is to find the directions that maximize the amount of explained mutual information (MI) between stimulus and response, which corresponds to maximization of the Kullback Leibler divergence between the two distributions. For the single filter model, MI is given by

$$\mathrm{MI}(\hat{\mathbf{k}}) = \int_X \mathrm{d}x P_{\hat{\mathbf{k}}}(x|y=1) \log_2 \left[\frac{P_{\hat{\mathbf{k}}}(x|y=1)}{P_{\hat{\mathbf{k}}}(x)} \right] \tag{1}$$

with $P_{\hat{\mathbf{k}}}$ denoting the distribution of projections x onto the filter estimate $\hat{\mathbf{k}}$.

For comparison, we also applied the MID algorithm using the numeric code available from `http://cnl-t.salk.edu` to our data. It implements simulated annealing and early stopping to prevent local maxima and overfitting, respectively. We started the employed combination of gradient ascend and simulated annealing algorithm with different numbers of iterations ranging from 50 to the dimensionality of the linear filter. We found the number of iterations after which the amount of explained MI does not change to be 400 for simulations and 500 for neural data. The annealing parameters were the same as described in [2].

Fitting of the Poisson Generalized Linear Model

The Poisson GLM assumes that the spiking process is a conditionally inhomogeneous Poisson process with rate $r_i \equiv f(\mathbf{s}_i{}^{\mathrm{T}}\mathbf{k}), i = 1, 2, , ..., N$. The log-likelihood of observing a spike train given the model parameters $\Theta = \{\mathbf{k}, \mu\}$ is given by

$$L(\Theta) = \sum_{i=1}^{N} n_i \log(r_i) - \Delta \sum_{i=1}^{N} r_i + c \tag{2}$$

where n_i is the number of spikes in the ith time bin, Δ the bin width, and c a constant independent of Θ. For both simulations and neural recordings the bin width was small and the response essentially binary. The canonical inverse link for the Poisson GLM is the exponential and the maximum a posteriori (MAP) estimate is given by

$$L(\Theta) = \sum_{i=1}^{N} \left(\mathbf{s}_i{}^{\mathrm{T}}\mathbf{k} + \mu\right) - \Delta \sum_{i=1}^{N} \exp\left(\mathbf{s}_i{}^{\mathrm{T}}\mathbf{k} + \mu\right) - \lambda \frac{\mathbf{k}^{\mathrm{T}}\mathbf{k}}{2} \tag{3}$$

where the regularization term on the right-hand side prevents overfitting.

The problem in Eq. (3) is convex and can be solved by standard gradient-ascent algorithms. Similarly to the CbRF method we used a trust region Newton algorithm to find the parameters of the model and the hyperparameter λ is found by maximizing MI between stimulus and response in a 5 fold cross-validation setting. We also test the log-likelihood as optimization criterion. However, MI yielded significantly better results for both simulations and neural recordings.

Reverse Correlation Receptive Field Estimation

The STA is the mean stimulus preceding a spike given by

$$\hat{\mathbf{k}}_{\text{STA}} \propto \langle \mathbf{s} r \rangle_{r=1} , \tag{4}$$

with $\langle\cdot\rangle_{r=1}$ denoting expectation over the spike-eliciting stimulus ensemble. Without loss of generality, it is assumed that $\langle \mathbf{s} \rangle = \mathbf{0}$. Second-order correlations in the stimulus can be removed by decorrelating Eq. (4) using the inverse of the autocovariance matrix yielding

$$\hat{\mathbf{k}}_{\text{decorr}} \propto \left\langle \mathbf{s}\mathbf{s}^{\text{T}} \right\rangle^{-1} \langle \mathbf{s} r \rangle . \tag{5}$$

This is mathematically equivalent to least squares linear regression.

Inversion of the auto-covariance matrix may result in an overamplification of high-frequency noise. To avoid such overfitting along undersampled dimensions we used a regularization scheme based on ridge regression yielding

$$\hat{\mathbf{k}}_{\text{ridge}} \propto \left\langle \mathbf{s}\mathbf{s}^{\text{T}} + \lambda \mathbf{I} \right\rangle^{-1} \langle \mathbf{s} r \rangle , \tag{6}$$

with a $D \times D$ identity matrix $\mathbf{I}$, regularization parameter $\lambda \geq 0$, ranging from an unregularized solution ($\lambda = 0$) to the STA ($\lambda \to \infty$) [3].

In a Bayesian interpretation, the ridge regression modification is equivalent to bias $\mathbf{k}$ towards solutions that are more probable with respect to a given multivariate prior distribution, which is Gaussian in this case, resulting in more sparse solutions. We used the regularization parameter that yielded the highest MI between stimulus and response (see Eq. (1)) in 5-fold cross-validation setting.

We also tested a regularization scheme based on singular value decomposition of the auto-covariance matrix. This form of the STA is also known as normalized reverse correlation (NRC, [4]). However, we found that ridge regression produces significantly better estimates for both simulated data and neural recordings.

Bias in the STA due to higher-order correlations in the stimulus ensemble

To investigate robustness of the different methods to higher-order correlations in the stimulus ensemble responses r were simulated using a LNP model with an exponential nonlinearity of the form [5]

$$r = \left\| \mathbf{s}^{\text{T}} \mathbf{k} \right\|_{+}^{p} \tag{7}$$

where $\|\cdot\|_{+}$ denotes half wave rectification (using only the positive projections of the stimuli onto the linear filter $\mathbf{k}$) and $p \in \mathbb{N}^{+}$ is the order of the nonlinearity. The filter estimate computed by the STA method (cf. Eq. (4)), setting $p = 2$, is given by

$$\hat{\mathbf{k}} \propto \left\langle \mathbf{s} \left\| \mathbf{s}^{\text{T}} \mathbf{k} \right\|_{+}^{2} \right\rangle_{+} , \tag{8}$$

denoting by $\langle \cdot \rangle_+$ the expectation over all stimulus examples with $r > 0$, and its ith component is

$$\hat{k}_i \propto \sum_j \sum_k k_j k_k \langle s_i s_j s_k \rangle_+. \tag{9}$$

Hence, $\hat{k}_i$ depends on third-order correlations $\langle s_i s_j s_k \rangle_+$ and may assume non-zero values even if s_i does not lie in the dimensional support of the true linear filter ($k_i = 0$). For $q > 2$, also correlations of higher order contribute to $\hat{k}_i$.

References

1. Adelman TL, Bialek W, Olberg RM (2003) The information content of receptive fields. Neuron 40: 823–833.

2. Sharpee T, Rust NC, Bialek W (2004) Analyzing neural responses to natural signals: maximally informative dimensions. Neural Comput 16: 223–250.

3. Machens CK, Wehr MS, Zador AM (2004) Linearity of cortical receptive fields measured with natural sounds. J Neurosci 24: 1089–1100.

4. Theunissen FE, David SV, Singh NC, Hsu A, Vinje WE, et al. (2001) Estimating spatio-temporal receptive fields of auditory and visual neurons from their responses to natural stimuli. Network 12: 289–316.

5. Christianson GB, Sahani M, Linden JF (2008) The consequences of response nonlinearities for interpretation of spectrotemporal receptive fields. J Neurosci 28: 446–455.
